# Alpha-synuclein in Parkinson’s disease and other synucleinopathies: from overt neurodegeneration back to early synaptic dysfunction

**DOI:** 10.1038/s41419-023-05672-9

**Published:** 2023-03-01

**Authors:** Paolo Calabresi, Alessandro Mechelli, Giuseppina Natale, Laura Volpicelli-Daley, Giulia Di Lazzaro, Veronica Ghiglieri

**Affiliations:** 1 Sezione di Neurologia, Dipartimento di Neuroscienze, Facoltà di Medicina e Chirurgia, Università Cattolica del Sacro Cuore, Rome, 00168 Italy; 2 Neurologia, Fondazione Policlinico Universitario Agostino Gemelli IRCCS, Rome, 00168 Italy; 3 Dipartimento di Scienze Mediche e Chirurgiche, Istituto di Neurologia, Università “Magna Graecia”, Catanzaro, Italy; 4 Center for Neurodegeneration and Experimental Therapeutics, University of Alabama at Birmingham, Birmingham, AL 35294 USA; 5 Università Telematica San Raffaele, Rome, 00166 Italy

**Keywords:** Parkinson's disease, Long-term potentiation

## Abstract

Although the discovery of the critical role of α-synuclein (α-syn) in the pathogenesis of Parkinson’s disease (PD) is now twenty-five years old, it still represents a milestone in PD research. Abnormal forms of α-syn trigger selective and progressive neuronal death through mitochondrial impairment, lysosomal dysfunction, and alteration of calcium homeostasis not only in PD but also in other α-syn-related neurodegenerative disorders such as dementia with Lewy bodies, multiple system atrophy, pure autonomic failure, and REM sleep behavior disorder. Furthermore, α-syn-dependent early synaptic and plastic alterations and the underlying mechanisms preceding overt neurodegeneration have attracted great interest. In particular, the presence of early inflammation in experimental models and PD patients, occurring before deposition and spreading of α-syn, suggests a mechanistic link between inflammation and synaptic dysfunction. The knowledge of these early mechanisms is of seminal importance to support the research on reliable biomarkers to precociously identify the disease and possible disease-modifying therapies targeting α-syn. In this review, we will discuss these critical issues, providing a state of the art of the role of this protein in early PD and other synucleinopathies.

## Facts

- Alpha-synuclein aggregates perturb dopaminergic transmission and induce presynaptic and postsynaptic dysfunctions.
- Distinct cell types can be differentially affected by alpha-synuclein aggregates over the course of the disease in preclinical studies.
- Immune response to alpha-synuclein misfolding contributes to disease progression.
- Parkinson’s Disease (PD), Dementia with Lewy Bodies (DLB), Multiple System Atrophy (MSA), Pure Autonomic Failure (PAF) and REM sleep Behavior Disorder (RBD) show synuclein-related neuroinflammation and share clinical, neurochemical and morphological features.
- New therapies targeting alpha-synuclein include passive and active immunization and modulators of protein aggregation.

## Main text

The discovery of the critical role of α-synuclein (α-syn) in the pathogenesis of Parkinson’s disease (PD) was postulated more than twenty years ago [1, 2] when genetic forms of PD were described. Abnormal aggregates of α-syn, such as Lewy bodies (LB) and Lewy neurites, and glial cell inclusions have been then implicated in several sporadic neurodegenerative diseases termed α-synucleinopathies, including idiopathic PD, Dementia with Lewy bodies (DLB), Multiple systems atrophy (MSA), Pure autonomic failure (PAF) and REM sleep behavior disorder (RBD) (see Table 1) [3–5]. However, many critical questions remain regarding its physiological role, as well as a successful strategy to target this protein to prevent neurodegenerative disease.

**Table 1:** Other synucleinopathies.

Main features
**Dementia with Lewy bodies (DLB)**
• DLB and Parkinson’s disease-dementia (PDD) are synuclein-related diseases that share many clinical, neurochemical and morphological features.
• The differential diagnosis between these disorders is based on a distinction between the time of onset of motor and cognitive symptoms. Dementia often precedes parkinsonism in DLB, while the onset of cognitive impairment follows motor symptoms in PDD.
• Diffuse cortical and subcortical aggregations of α-syn (Lewy bodies, LB) plus β-amyloid and tau pathologies can be present in both conditions.
• Clinical and neuropathological findings suggest that DLB is characterized by a distinct regional spreading pattern of LB, a less robust response to dopaminergic therapy and a high prevalence of Alzheimer-like pathology at *postmortem* examination.
**Multiple system atrophy (MSA)**
• MSA is an adult-onset sporadic α-synucleinopathy, with symptoms of parkinsonism, cerebellar ataxia and a variable combination of autonomic failure.
• MSA patients are classified as MSA-P, with predominant levodopa-unresponsive parkinsonism caused by striatonigral degeneration, and MSA-C, with cerebellar ataxia associated with olivopontocerebellar atrophy.
• MSA is defined by neuronal loss and abundant filamentous α-syn inclusions selectively found in oligodendrocytes; α-syn filaments from the brains of patients with MSA and DLB have different conformations, suggesting that distinct strains characterize these conditions.
• Transgenic and virus-based models overexpressing human α-syn in oligodendrocytes using different promoters have been generated to recapitulate the disease.
• Ongoing clinical trials target α-syn through immunotherapy approaches, inhibition of oligomerization and aggregation of this protein.
**Pure autonomic failure (PAF)**
• PAF is a rare and sporadic neurodegenerative disorder of the autonomic nervous system, clinically characterized by orthostatic hypotension. Autonomic failure may also manifest as genitourinary, bowel, and thermoregulatory dysfunctions. REM behavior disorder and hyposmia are also common features in patients with PAF.
• Pathologically, PAF is characterized by predominantly peripheral deposition of α-syn involving the sympathetic ganglia and peripheral autonomic nerves. LBs, though, can also be found in central nervous system structures such as substantia nigra and locus coeruleus.
• About one-third of PAF patients show phenoconversion to a central α-synucleinopathy with motor or cognitive involvement such as PD, DLB, or MSA within 4 years from the diagnosis.
• Recently, a mouse model of PAF has been generated with α-syn preformed fibrils (PFFs) injection into the stellate and celiac ganglia, generating a spreading of the α-syn pathology only through the autonomic nervous system bidirectionally.
**REM behavior disorder (RBD)**
• RBD is a REM sleep parasomnia first described in 1986 and characterized by the loss of physiological muscle atonia typical of REM sleep.
• In recent years, follow-up studies have shown that most RBD patients will develop an overt α-synucleinopathy over time, with a rate of phenoconversion of about 75% after 12 years from diagnosis.
• To date, no reliable candidate biomarkers can predict phenoconversion, its timing, and the phenotype of the α-synucleinopathy the patient will develop.

In the first part of the review, we report new evidence on the current knowledge on α-syn physiological function in cellular structures and organelles, in particular focusing on the synaptic sites. We then discuss how pathological α-syn modulates dopaminergic transmission and its potential impact on other susceptible neuron subtypes.

In the second part of the review, the emerging role of α-syn-mediated neuroinflammation is presented and discussed as a critical factor leading the way to the onset of neuronal death pathways and its potential impact on synapse loss. Moreover, we provide an overview of the recent findings suggesting that α-syn-induced early synaptic dysfunctions may precede overt neurodegeneration as a critical mechanism causing prodromal PD symptoms.

We also provide an overview of the clinical evidence that measures of different forms of α-syn, alone or combined with other molecules, may represent reliable biomarkers of disease initiation and progression in PD and other synucleinopathies. Finally, we summarize clinical trials on disease-modifying therapies targeting α-syn in these neurodegenerative disorders.

## Alpha-synuclein structure and physiological function

**Fig. 1:**
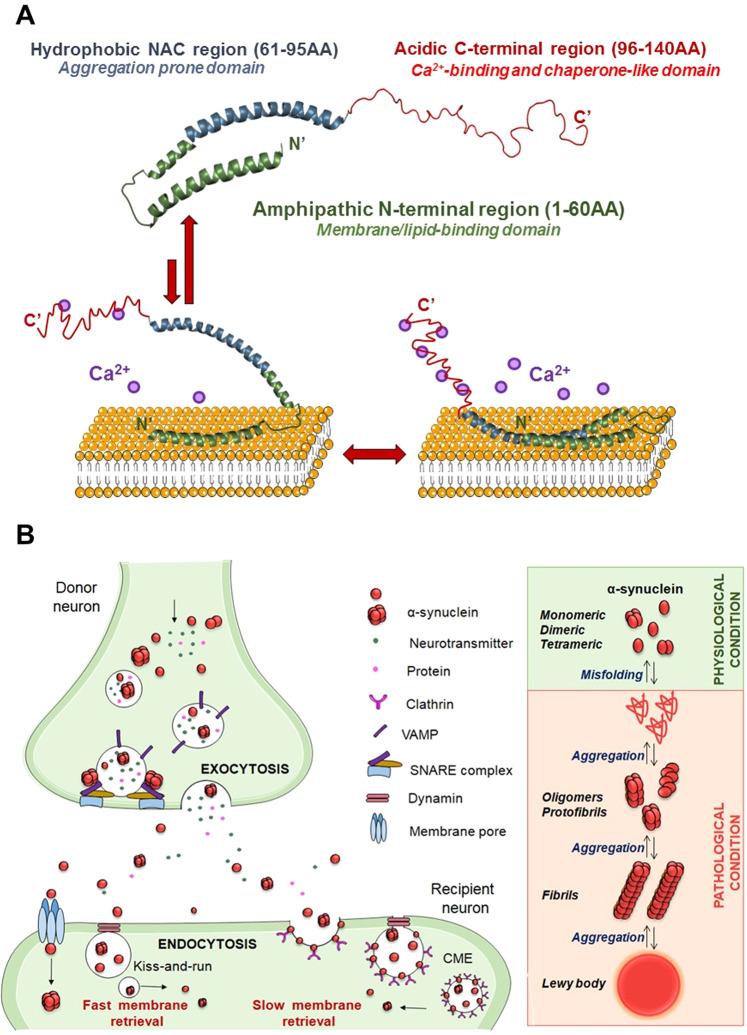
Modular structure of α-syn and its dynamic interactions with membrane lipids in physiological and pathological conditions. **A** Alpha-syn is structurally characterized by three modular regions: the N-terminal (green), characterized by amphipathic repetitions, is responsible for interactions with membranes; the hydrophobic NAC (blue), is relevant for aggregation, and the acidic C-terminal (red), is involved in Ca^2+^ binding and chaperon-like activity. In the presence of low concentrations of Ca^2+^, α-syn is stably anchored to the lipid surface via its N-terminal region only. With higher Ca^2+^ concentrations, also the NAC region, but not the C-terminus, shows lipid-binding properties, suggesting that it plays a different role in modulating the affinity of α-syn for cellular membranes. **B** Left panel: in control conditions, α-syn is important for regulating protein and neurotransmitter release by promoting SNARE complex formation and vesicle docking during the exocytosis process. Extracellular α-syn can affect recipient neurons by fast (i.e., kiss-and-run mode) and slow (i.e., clathrin-mediated endocytosis, CME) modes of membrane retrieval, as several endocytotic pathways can coexist in a single synapse. Interestingly, α-syn is associated with the CME of synaptic vesicles due to its role in sensing and stabilizing the curved membranes. Right panel: under physiological conditions (green), α-syn exists in different conformations balance between unstructured soluble monomeric and tetrameric forms. Under pathological conditions (light red), when the balance between α-syn generation and clearance is disrupted, α‐syn aggregates into oligomers, protofibrils, and fibrils, which further bring to the formation of protein inclusions called LB.

A-syn is a small (14 KDa) acidic protein expressed in neurons of the central and peripheral nervous system as well as in blood cells and other tissues [6]. A-syn has long been defined as a ‘natively unfolded’ monomer; however, it has been demonstrated that endogenous α-syn naturally occurs in large part as a folded tetramer of about 58 KDa with little or no amyloid-like aggregation potential [7]. The two forms coexist, but an imbalance in the tetramer: monomer ratio can lead to the preponderance of pro-aggregating forms. A-syn can be subdivided into three main regions, each responsible for different molecular and biological properties [8]. The amino acid residues 1–60 represent the N-terminus, which is characterized by amphipathic repetitions that tend to form an α-helix structure. This portion of the protein is crucial for the capacity of α-syn to interact with membranes [9]. Residues 61–95 constitute the non-amyloid-ß component (NAC region), identified as the most aggregation-prone region. The final C-terminus (96–140) is negatively charged and is involved in Ca^2+^ binding and chaperone-like activity [10] (Fig. 1A). A recent study showed that the binding of Ca^2+^ to the C terminus of α-syn also regulates its binding to synaptic membranes [11]. In PD, LBD and PAF, α-syn adopts a pathological β-sheet conformation that recruits additional monomers to form oligomers and amyloid fibrils. The inclusions localize to axons, called Lewy neurites, or the neuron soma, called LB [8, 11]. In MSA, α-syn aggregates form mainly glial cytoplasmic inclusions, also called Papp-Lantos bodies, argyrophilic inclusions in oligodendrocytes cytoplasm. Neuronal cytoplasmic inclusions are also found, but with distinct distributions as compared to LB, being observed in the putamen, pontine nuclei, and inferior olivary nuclei [12].

A-syn pathology can be observed in in vitro and in vivo models through the introduction of pathological α-syn seeds in the form of preformed fibrils (PFFs) from recombinant α-syn monomers [13], α-syn obtained from brain samples of patients [14] or rodents carrying α-syn mutations [15]. A-syn aggregates can also be obtained via viral vector-mediated overexpression of wild-type α-syn [16]. The different forms of α-syn aggregates or mutant α-syn might initiate distinct processes in terms of perturbation of physiological function or damaging effect. However, most molecular mechanisms triggered by these pathological forms of α-syn might share common pathways converging to similar synaptic dysfunctions.

The interaction of monomeric α-syn with its fibrillar form is still debatable. Recently, a transient interaction of α-syn monomers via their positively charged N-terminus with the negatively charged flexible C-terminal ends of the fibrils was demonstrated [17]. These intermolecular interactions result in an unfolding process and consequent exposure of the aggregation-prone components of the protein. This condition represents a prerequisite for protein aggregation that leads to rapid multiplication of α-syn, catalyzing the generation of new amyloids from monomers on their surface, a process called secondary nucleation. Oligomers produced during secondary surface nucleation induce the permeabilization of cell membranes through to their lipophilic properties. Associated with these events, higher basal levels of cytosolic Ca^2+^, increased Ca^2+^ influx upon stimulation, and delayed recovery of Ca^2+^ levels in neurons are then observed since the early phases, leading to multiple Ca^2+^ homeostasis alterations that persist with disease progression [18].

Native α-syn is present in synaptic terminals, in the nucleus of neuronal cells [19], mitochondria [20], endoplasmic reticulum (ER) [21], Golgi apparatus (GA) [22], and in the endolysosomal system [23]. However, its physiological function in each subcellular compartment is only partially understood.

A-syn is mainly present at presynaptic sites where it interacts with proteins involved in the release and reuptake of neurotransmitters [24]. Moreover, α-syn is involved in sensing and stabilizing curved membranes [25] and regulating the synaptic vesicle pool and trafficking [26]. In this regard, α-syn has a role in the assembly of the Soluble N-ethylmaleimide Attachment protein REceptor (SNARE) complex, which consists of a set of presynaptic proteins that mediate different phases of the exocytosis process [24] (Fig. 1B). Results obtained from mice with either knockout or overexpression of the α-syn gene support the concept of a regulatory function of α-syn in exocytosis and neurotransmitter release. A recent study showed that α-syn interacts with vesicle-associated membrane protein 2 (VAMP2) where it acts as a brake on synaptic vesicle exocytosis [27]. The lack of physiological α-syn has been associated with a reduction in the number of synaptic vesicles of distal pools [28], whereas its overexpression has been shown to increase the number of vesicles docked to the membranes [29]. For these features, α-syn can be essential in preventing a vesicle collapse during kiss-and-run exocytosis and improves fast membrane retrieval mechanisms. Given the coexistence of different endocytosis dynamics in the same synapse, clathrin-mediated endocytosis (CME) or fast membrane retrieval pathways operate according to synaptic activity. Neurons that fire with high frequencies may need fast or ultrafast membrane-saving mechanisms to maintain the structure of the presynaptic terminal, compared with low-frequency firing neurons, whose endocytosis activity can be sufficiently sustained by CME. Interestingly, synucleins may interact with these processes, as they are necessary for the rapid internalization of synaptic vesicle membranes, as demonstrated by the observation that the absence of all three synuclein isoforms, α-, β- and γ-, results in slowed endocytosis [30].

This possible interaction is particularly relevant in cells with pacemaker activity, like the dopaminergic neurons of the substantia nigra pars compacta (SNpc), the neuronal population most severely affected in PD [31]. The continuous cycles of exo- and endocytosis that these cells undergo throughout their life require fast and highly efficient molecular machinery to maintain intact presynaptic vesicle homeostasis. When fast mechanisms fail, CME may temporarily manage to guarantee a certain level of vesicle supply that might not be sufficient for the demands of high-frequency firing neurons. In that context, α-syn could have a regulatory role in maintaining synaptic homeostasis upon intense neuronal activity.

If α-syn is involved in neuronal activity, could its sequestration into pathologic aggregates have implications on synaptic transmission? In particular, how does synaptic transmission influence the spread of abnormal forms of α-syn? In vitro and in vivo microdialysis experiments showed that ∼70% of extracellular α-syn derives from neuronal activity-dependent pathways [32].

Several post-translational modifications, such as phosphorylation, can affect α-syn folding and aggregation, playing a critical role in PD and other synucleinopathies. Less than 5% of the soluble, monomeric α-syn is phosphorylated under physiological conditions. Conversely, approximately 90% of α-syn is phosphorylated in LBs, suggesting a close relationship between phosphorylation at S129 and α-syn aggregation in PD and other synucleinopathies [33]. Moreover, phosphorylation of other residues localized at the C-terminal region of the protein has been associated with these diseases [33].

## A-syn and dopaminergic transmission

Since α-syn is implicated in sporadic and familial forms of PD, many studies have investigated its role in modulating dopamine (DA) neurotransmission. Mutant mice lacking α-syn show a reduced DA striatal content and inhibition of DA-dependent motor response to amphetamine, which enhances motor activity through the DA active transporter (DAT)-dependent mechanism [34, 35]. Moreover, animal models’ data strongly agree that overexpression of α-syn leads to a direct insult to the dopaminergic system, reported as a decrease in tyrosine hydroxylase (TH) activity and expression within the striatum [36]. However, one drawback of using transgenic mice overexpressing α-syn as a model for PD is that it is not clear if phenotypes are caused by α-syn overexpression and more normal α-helical α-syn at membranes or increased formation of pathological α-syn. Therefore, models that more directly recapitulate the formation of pathological α-syn assemblies better reflect the impact of abnormal α-syn in DA transmission.

Exposure of neurons to α-syn oligomers increases Ca^2+^ intracellular levels, thus inducing DA release in striatal slices via multiple mechanisms [37]. When neuronal Ca^2+^ homeostasis is disrupted, ATP expenses for maintaining an electrochemical gradient across the membrane increase, enhancing neuronal vulnerability and altering neurotransmitter release. Slow Ca^2+^ oscillations in SNpc DA neurons trigger their typical slow tonic firing, inducing an oscillation in membrane potential [38, 39]. These oscillations also cause Ca^2+^ influx into the mitochondria, which leads to oxidative phosphorylation and the production of ATP [40]. Elevated mitochondrial Ca^2+^ and oxidative phosphorylation promote further α-syn aggregation [41]. Thus, the Ca^2+^ oscillations in DA neurons combined with pathological α-syn aggregates can initiate a toxic cascade leading to neurodegeneration.

In physiological conditions, α-syn also controls the storage of DA into synaptic vesicles by interacting with vesicular monoamine transporter 2 (VMAT2) expression and activity in nigral neurons. Since VMAT2 is essential in reducing harmful oxidative effects of DA metabolites in the cytosol, an impairment in the sequestration of DA into the synaptic vesicles caused by abnormal α-syn may represent one of the earliest events in the degeneration of dopaminergic neurons [42]. The relationship between overexpression of α-syn and DAT activity and trafficking has been postulated, suggesting a positive correlation between α-syn levels and the reduction in DA uptake in the dorsal striatum [43].

Taken together, these data suggest that long-term overexpression of α-syn decreases DA neurotransmission at different levels by reducing TH and VMAT2 expression and activity, increasing DAT-mediated DA efflux, and reducing its uptake.

## Neurodegeneration and α-syn: a question of selective cell-type vulnerability?

Accordingly, neurons with extensive axonal arborizations and high requirements for mitochondrial activity, such as DA neurons, may be more susceptible to α-syn aggregation [41]. A-syn plays a direct role in mitochondrial activity [44, 45]. In particular, pathological α-syn mediates functional mitochondrial failure, disruption of mitochondrial morphology [46], impairment of complex I function [47], mitochondria accumulation, and decreased basal mitochondrial oxygen consumption rate [45]. There is also evidence that nitrosative and oxidative stress causes abnormal protein accumulation by impairing the ubiquitin-proteasome system [48], leading to a vicious circle of oxidative cellular damage, toxic α-syn accumulation, and neuronal death. In particular, increased mitochondrial oxidative stress in fibroblast-derived induced pluripotent stem cells, differentiated into human nigral DA neurons, triggers a DA-dependent toxic cascade, leading to oxidized DA accumulation and reduction in glucocerebrosidase enzymatic activity, lysosomal dysfunctions, and α-syn accumulation [49]. Besides these events, age‐related redox changes within nigral neurons are more evident within the ventral tier, suggesting that increased redox dyshomeostasis may underlie the selective vulnerability of this nigral subregion [50].

While α-syn-related damage to mitochondrial activity is a common mechanism for virtually all cell body types, α-syn aggregates are not found randomly in the central nervous system (CNS) of PD patients [51], implying an increased susceptibility of specific brain areas to LB formation. A combination of structural (i.e., extensively branched axons with a high number of neurotransmitter release sites in spatially distributed networks) and functional features (i.e., broad spikes, slow pacemaking activity, low intrinsic Ca^2+^ buffering and cytosolic Ca^2+^) seems to better identify the neurons most vulnerable in PD [31]. These neuron-specific aspects of cell function are energetically expensive. It has been proposed that the limited energetic reserve this unique axonal architecture imposes on DA nigral neurons is a key factor in their susceptibility. Under normal circumstances, this high energy demand can be managed by neurons. However, any situation that perturbs the balance between energy production and demand — such as mitochondrial dysfunction or oxidative stress — would lead to functional failure and, eventually, cell death [52].

Therefore, the factors regulating the spread of pathogenic α-syn forms remain a matter of debate due to an incomplete understanding of how the vulnerability works in the context of spread. Recently, by using a quantitative pathology mapping approach in the mouse brain, the spatiotemporal pattern of spread has been clearly associated with two main factors: anatomical connectivity and endogenous α-syn expression in a given area [53].

In line with this preclinical study, results provided by the PD progression markers initiative (PPMI) cohort indicate three spatial modes of disease spread associated with a typical, a severe, and a milder progression. The “typical” progression corresponds to the Braak staging scheme [54]. The more severe expression appears to involve earlier propagation of the disease to limbic and prefrontal areas, possibly accounting for dementia, mood disturbances, and hallucinations seen in more severe/advanced cases of PD. In the milder phenotypes, the tremor-dominant forms with less cognitive impairment, dopaminergic innervations are relatively preserved for more extended periods. This tripartite dissociation of clinical severity accords with hierarchical classification schemes based on clinical features [55].

An interesting issue regarding the relationship between LB pathology and the occurrence of clinical parkinsonism is the description of incidental Lewy body disease features. In fact, neuropathological studies showed a distribution pattern of α-syn-related pathology also in those aged individuals without a history of parkinsonism [56]. This evidence supports the hypothesis that nigral neuronal loss and diminished TH immunoreactivity precede the appearance of local α-syn pathological conditions, suggesting that other mechanisms, or yet undetected conformations of α-syn, such as monomers and/or oligomers, determine nigral neuronal dysfunction during preclinical stages [57].

## A-syn and activation of the immune system in PD

A growing body of evidence suggests that inflammation participates in the pathophysiology of PD and other synucleinopathies at multiple levels and with different mechanisms, ultimately contributing to disease progression.

**Fig. 2:**
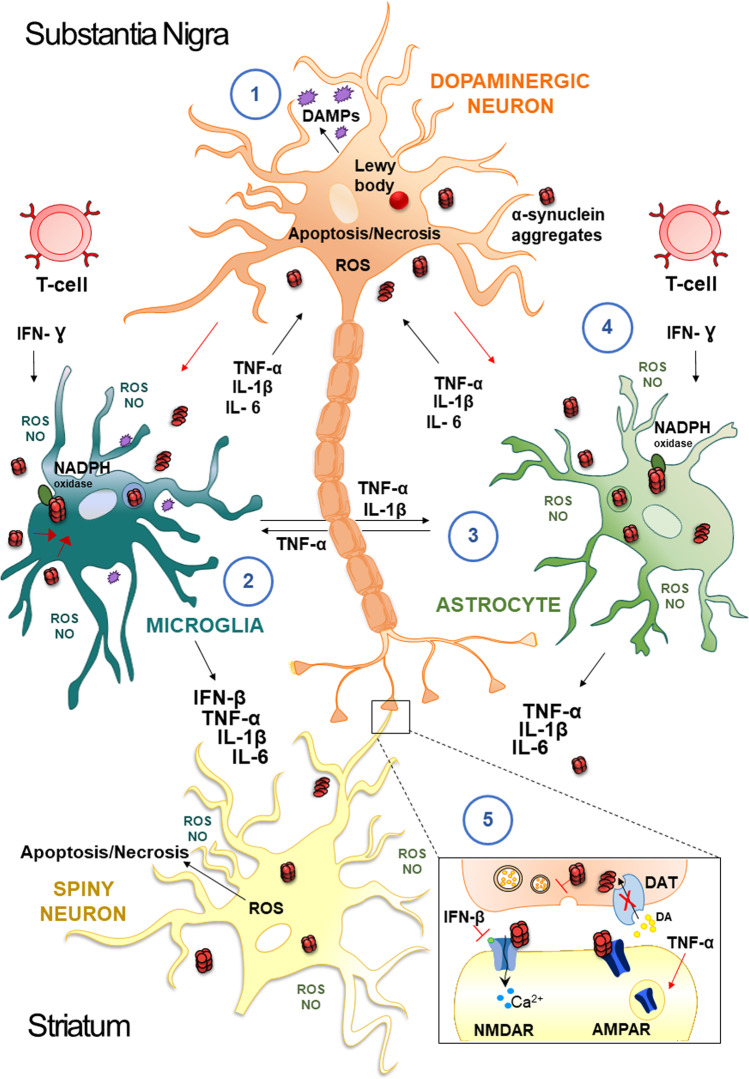
Mechanisms underlying inflammation in PD. Neuropathological hallmarks of PD are the presence of intracellular inclusions containing α-syn aggregates and the death of dopaminergic neurons in the SNpc of the midbrain. Damage-Associated Molecular Patterns (DAMPs), are endogenous danger molecules released from damaged or dying cells, resulting in microglia activation and persistent neuroinflammation (1). Extracellular aggregates of α-syn released by neurons activate glial cells; the internalization of α-syn is followed by activation of NADPH oxidase with the production of ROS/NO, and by the release of proinflammatory cytokines (TNF-α, IL-1β, IL-6, IFN-β) (2). In addition, activated-microglial cells produce mediators such as TNF-α and IL-1β that activate astrocytes, which in turn secrete other cytokines (TNF-α, IL-1β, IL-6) (3). These factors act on dopaminergic neurons of the SNpc and spiny neurons of the striatum and further activate microglia, amplifying the inflammatory response in a positive feedback loop. In particular, IFN-γ is secreted by T cells and is a cytokine involved in the death of DA neurons in the development of PD, which is responsible for enhancing the activation of the surrounding glial cells (4). In this scenario, soluble immune molecules can influence the modulation of synaptic transmission and plasticity (5). For example, INF-β influences NMDAR-mediated synaptic currents in spiny neurons. Conversely, TNF-α drives the internalization of AMPARs and reduces corticostriatal synaptic strength and results in a preferential removal of Ca^2+^-permeable AMPARs.

A-syn gene (SNCA) missense mutations lead to the accumulation of α-syn and activation of microglia, neuroinflammation, and degeneration of the striatal neurons [58]. Inflammatory mediators, such as reactive oxygen species (ROS), nitric oxide (NO), tumor necrosis factor (TNF)-alpha(α), and interleukin (IL)-1beta(β), derived from non-neuronal cells, are known to modulate the progression of neuronal cell death in PD [59, 60] (Fig. 2).

The main events of neuroinflammation in which α-syn seems to take an active part are the activation of microglia, the adaptive immune response, and the mitochondrial dysfunctions [61]. Microglia represents the main resident cellular population with immunological functions in the brain. These cells maintain a deactivated profile while probing the surrounding microenvironment, scavenging lipids, cellular debris, protein aggregates, and secreting factors essential for the survival and activity of neurons and astrocytes [62]. Several studies, both in vivo and in vitro, showed that α-syn could activate microglia, thus generating a pathological inflammatory stimulus with different intensities of response depending on the α-syn isoforms and PD-related gene mutations [63]. Mutant monomeric α-syn triggers a strong immune system response at concentrations, in which wild-type α-syn does not cause an immune activation [64]. Regarding α-syn aggregates, all oligomeric α-syn species induce a dramatic immune cell activation, showing a correlation between the molecular weight of oligomers and the intensity of the response, with α-syn fibrils producing the most powerful activation of monocytes [64].

Given the association between inflammation and higher risk for PD [65, 66], these data suggest that α-syn pathology and inflammatory state play a synergistic role in the excessive immune system response contributing to the worsening of the PD state. Accordingly, cytokines levels, like IL-1β, TNF-α, IL-6, TGF-β, and IFN-γ, were found elevated in CSF and serum of PD patients, suggesting an α-syn-mediated microglial activation [67]. The microglia activation and the subsequent immune response might spread across the brain along with the α-syn inclusions, implying the participation of the innate immune system in the disease course [68]. Microgliosis may also contribute to the phagocytosis of injured synapses, as shown in Alzheimer’s disease (AD) [69].

Concerning the involvement of the adaptive immune system in PD neuropathology, data obtained from human studies and animal experiments have shown infiltrations of both CD8+ and CD4+ T cells in the SNpc of PD subjects [70, 71] (Fig. 2), which may eventually lead to adaptive immune responses [72].

However, the relationship between adaptive immune responses and PD clinical history in human subjects has been only recently explored, as documented by an important study investigating α-syn-specific T cell reactivity in two different cohorts of PD patients with different disease duration [73]. T cells were most reactive to α-syn in patients closer to the diagnosis, and their response seems to lower over the years. Interestingly, T cells were reported to respond to α-syn epitopes found in both extracellular native α-syn and fibrilized α-syn [74].

Concerning the role of B cells in α-syn-related pathology, findings are still controversial since some studies report no alterations while others describe a decrement of these cells in the peripheral blood population of PD patients [75]. Nonetheless, peripheral IgG^+^ B cells producing anti-α-syn antibodies were isolated from patients, and three different antibodies displayed α-syn aggregation inhibiting properties, suggesting a protective role of IgG in PD pathogenesis [75].

## Inflammation and other synucleinopathies

### Dementia with Lewy bodies

DLB is characterized by a combination of clinical features, including recurrent visual hallucinations, cognitive fluctuations, and motor symptoms of parkinsonism. Although several studies have investigated inflammation in PD, there is evidence showing that inflammation also plays an important role in DLB [76]. Pathological findings and preclinical studies suggest that both α-syn and amyloid/tau pathology play a role in initiating neuroinflammatory processes in DLB. Similarly, PET imaging and blood biomarkers support an increase in cerebral and peripheral inflammation in the early phases of DLB, while these features are reduced with disease progression [77]. Moreover, it has been suggested that in DLB, as well as in PD, markers of inflammation may play a sentinel role in the early phase of the disease. Indeed, the interaction between inflammation and α-syn-related and AD pathology composes the complex pathological scenario for the appearance of clinical DLB.

### Multiple system atrophy

MSA is a neurodegenerative disease characterized by parkinsonism, ataxia, and dysautonomia. Histopathologically, the hallmark of MSA is the abnormal accumulation of α-syn within oligodendroglial cells, leading to neuroinflammation, demyelination, and neuronal death.

It has been reported an astrocytic along with microglial activation in the MSA brain [78]. A recent study has also shown that even if a significant change in brain cytokines, typical microglial products, cannot be detected, the analysis of mRNA shows a significant change in the expression of a subset of inflammation-associated genes. This finding confirms that an inflammatory response is initiated in MSA [79]. At present, there is no disease-modifying treatment for MSA. Thus, it is possible that targeting inflammation-related processes might limit the disease progression.

### REM sleep behavior disorder

RBD can precede synucleinopathies, and may represent a prodromal phenotype of PD. The role of neuroinflammation in RBD and the association of this disorder with peripheral blood inflammatory markers is a matter of debate. In fact, a recent study has revealed increased microglial activation in the substantia nigra in association with reduced nigrostriatal dopaminergic activity [80]. However, this finding has been questioned by a subsequent study that did not provide evidence supporting the role of peripheral inflammation in RBD [81]. A more recent prospective case-control study showed an association between peripheral blood monocytes and brain immune and dopaminergic changes in RBD, supporting the occurrence of an interplay between peripheral inflammation and the brain during the disease. In particular, the study revealed that the expression levels of Toll-like receptor 4 on blood monocytes in RBD patients are positively correlated with nigral immune activation measured by 11C-PK11195 PET and negatively correlated with putaminal 18F-DOPA uptake [82].

### Pure autonomic failure

Autonomic dysfunction is a frequent and common disturbance in synucleinopathies, often appearing in the early stage of the disease. Among the α-synucleinopathies, PAF is characterized by slowly progressive autonomic failure without motor dysfunctions. Postmortem studies show LB localization in the autonomic nuclei and substantia nigra [83]. Interestingly, a recent preclinical study proposed a new model in which inoculation of α-syn PFFs into the stellate and celiac ganglia induces bidirectional spreading of α-syn from the CNS to the peripheral organs via their autonomic innervation [84]. Nevertheless, at present, no clear evidence regarding the role of central and peripheral inflammation in PAF pathophysiology has been provided.

## A-syn and early synaptic dysfunction

Evidence from both human and animal models has demonstrated that before clear signs of neurodegeneration appear in specific brain regions, other dysfunctions occur in the same neuronal circuits at a synaptic level in apparently intact cells. Indeed, being α-syn a presynaptic protein, any toxic effect or even a partial loss of function can disrupt synaptic functionality.

### Presynaptic dysfunctions induced by α-syn in PD

Current research is increasingly focusing on the earliest pathological synaptic changes induced by α-syn, as dysfunction of dopaminergic neurotransmission in PD was reported to precede Lewy pathology and neurodegeneration [57, 85]. Neuropathological evidence from PD patients shows that dopaminergic terminals in the putamen are lost significantly earlier than neuron soma [86]. This finding was also confirmed by PET studies that identified a threshold for VMAT2 and DAT loss in nigrostriatal terminals before any clinically noticeable manifestation of the disease [87]. Animal models of PD where α-syn was overexpressed via adeno-associated viral (AAV) vectors, or mice genetically modified to display α-syn fibril-induced formation, confirmed that loss of dopaminergic terminals occurs before any detectable neuron loss in SNpc [88–91].

A-syn aggregates at the axonal level lead to impaired microtubular transport from the cell body to terminals [92–94]. This event also impairs the transport to the soma of retrograde signaling molecules and endosomes from synaptic terminals, thus hindering the activation of transcription factors essential for survival and neurite outgrowth [95].

As described above, α-syn aggregates gradually impair the molecular machinery involved in DA release from presynaptic terminals [96, 97], possibly through interactions with oxidized DA that increase the chance for this protein to accumulate. Α-syn inclusions are also involved in the segregation of components of the presynaptic apparatus and neurotransmitter transporters, including DAT and VMAT2, ultimately initiating a vicious cycle of α-syn accumulation and deregulation of DA release [26].

### Postsynaptic dysfunctions induced by α-syn in PD models

The complex role of DA as a key modulator of synaptic plasticity was thoroughly appraised by previous studies on the regulatory effect of DA over striatal neurons’ activity [98, 99]. On this basis, a large body of research investigated if α-syn can be responsible for synaptic alterations before a significant reduction of DA levels occurs in manifest PD.

The possibility that α-syn toxic forms cause early alterations of synaptic plasticity in PD in a cell-type-dependent way was explored within the striatum. Among the various neuronal subtypes in this nucleus, striatal cholinergic interneurons (ChIs) attracted attention as important targets of SNpc neurons because of their synchronized activity as a crucial trigger for DA release from nigrostriatal terminals [100, 101]. ChIs also receive glutamatergic inputs from cortical areas, are involved in reward-related learning processes, and respond to stimuli that mainly arise from the thalamostriatal afferents [102–104]. Thus, impaired activity of these interneurons might have dramatic consequences on cognition and movement. Tozzi and colleagues investigated if pathological forms of α-syn could induce early synaptic dysfunctions in ChIs and their influence on cognitive and motor deficits in animal models of PD, with different degrees of endogenous DA levels [105]. Interestingly, a very low in vitro concentration of oligomeric α-syn (3 nM) induced loss of LTP in ChIs, but not in spiny projection neurons (SPNs), proving a cell type-specific and dose-dependent alteration of synaptic plasticity. A similar electrophysiological alteration was accompanied in in vivo models by motor and cognitive defects, and it was mechanistically attributed to α-syn oligomers directly targeting GluN2D-containing N-methyl-d-aspartate (NMDA) receptors, which are selectively expressed on ChIs, but not on SPNs. Thus, early dysfunction of the striatal cholinergic system induced by α-syn oligomers seems sufficient to initiate the impairment of cognitive and motor activity in the early phases of PD [105].

SPNs were also recently investigated for the first time for detecting early synaptic transmission alterations exerted by α-syn oligomers, combining multiple techniques like electrophysiology optogenetics, immunofluorescence, and molecular and behavioral analyses [106]. Higher nanomolar (30 nM) concentrations of α-syn oligomers reduce NMDA receptor-mediated synaptic currents and impair corticostriatal and thalamostriatal LTP in SPNs of both direct and indirect pathways in vitro [106]. A-syn oligomers were proven to selectively interfere with GluN2A, which is crucial for LTP induction in SPNs [107], by disturbing its localization at the postsynaptic site. Of interest, monoclonal antibodies against oligomeric forms of α-syn were sufficient to prevent the reduction of GluN2A levels in the postsynaptic compartment and the consequent loss of LTP.

In vivo intrastriatal injection of α-syn oligomers in experimental animals led to deficits in visuospatial learning, in association with reduced expression of GluN2A NMDA receptor subunit, indicating a selective targeting by α-syn oligomers both ex vivo and in vivo [106].

**Fig. 3:**
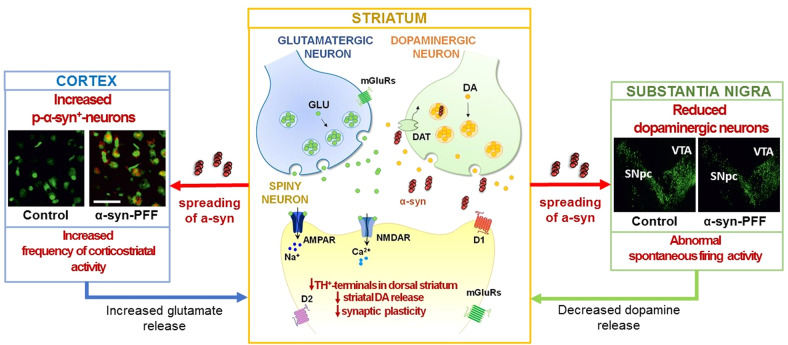
Presynaptic and postsynaptic dysfunctions induced by intrastriatal injection of α-syn-PFFs in the rat model. Left panel: in the cortical areas, a consistent proportion of p-α-syn^+^ neurons was detected in α-syn-PFFs-injected rats. Analysis of spontaneous synaptic currents indicates an increased frequency of the spontaneous excitatory postsynaptic current in target neurons of the dorsal striatum that brings to a state of hyperglutamatergic activity. Right panel: the SNpc in α-syn-PFF rats presents a reduced number of dopaminergic neurons as displayed by a decrease of TH^+^-immunofluorence, associated with an abnormal increase in spontaneous firing activity. Center panel: in the dorsolateral striatum, α-syn-PFFs injection leads to profound alterations of the corticostriatal long-term plasticity, in the SPNs. A significant decrease of TH^+^ fibers and a reduced release of endogenous dopamine from SNpc terminals are also observed.

Most recently, the concomitant pre- and postsynaptic changes induced by α-syn were investigated by analyzing the effects of intrastriatal α-syn PFFs on corticostriatal and nigral physiology. A specific α-syn-induced dysregulation of nigral neuron activity impairs, in a time-dependent manner, the two primary forms of striatal synaptic plasticity, long-term depression (LTD) and LTP, before overt neurodegeneration. The defective nigral and striatal neuronal activity was accompanied by a reduced release of DA and increased glutamatergic transmission [108] (Fig. 3). These studies demonstrated that striatal neuronal populations feature a concentration-dependent susceptibility to α-syn toxicity and that impairment of the functionality of the basal ganglia network in synucleinopathies should be conceived as a complex emergent phenomenon rather than a single-threshold event.

These preclinical findings might also have implications for therapies that clear α-syn aggregates (see below). These therapies are typically viewed as potentially protective against neuronal death, and require long-term assessments to reveal a beneficial disease-modifying effect. The possibility to directly restoring synaptic functioning could achieve an early benefit, especially if the treatment is delivered in the initial phases of the disease. Unfortunately, this approach, which is theoretically feasible, has not been explored yet in clinical trials.

### Synaptic dysfunctions in other synucleinopathies

Multiple system atrophy. MSA models are characterized by ectopic deposition of abnormal α-syn predominantly in oligodendrocytes. A recent study investigating MSA models with cognitive impairment demonstrated that α-syn was first expressed in oligodendrocytes but then accumulated in the cytoplasm of excitatory hippocampal neurons and their presynaptic nerve terminals [109]. These findings were associated with the onset of memory impairment. Interestingly, in the model, α-syn oligomers increased in the hippocampus and, in parallel, hippocampal dendritic spine density was decreased. Moreover, LTP was suppressed in CA1, CA3, and dentate gyrus. Accordingly, postmortem analysis of human MSA brain tissues showed that patients with memory impairment have more α-syn inclusions in excitatory hippocampal neurons along with α-syn oligomers than subjects without cognitive deficits [109].

Dementia with Lewy bodies. Progressive accumulation of α-syn in the presynaptic terminal has been strongly associated with the pathogenesis of DLB. While the precise mechanisms are not fully understood, changes in kinase pathways, particularly mitogen-activated protein kinase (MAPK) p38 may play a role. In particular, a recent study investigated the expression of the p38 family in the brains of α-syn overexpressing transgenic mice and patients with DLB. Immunohistochemical analysis revealed that p38 is associated with neurons and astroglial cells and localized to presynaptic terminals. However, in the α-syn transgenic mice, p38 levels were increased in astroglial cells, while in neurons, it localized in the neuronal cell bodies instead of the presynaptic terminals. A similar finding was observed in DLB patients, where p38 also colocalized with α-syn aggregates. These results suggest that α-syn might interfere with the p38 pathway and alter synaptic functioning in DLB [110]. This idea is further supported by a recent imaging study documenting a widespread cortical reduction of synaptic density in a cohort of DLB/PD with dementia subjects using in vivo [11 C]UCB-J PET [111].

REM sleep behavior disorder. Even though the cause of RBD is unknown, multiple lines of evidence indicate that abnormal inhibitory transmission underlies the disorder. In particular, a study on transgenic mice with deficient glycine and GABA transmission showed that the deficits in glycine- and GABA(A)-mediated inhibition are associated with RBD symptoms [112]. Moreover, another study on rats with genetically-induced alterations of the inhibitory neurons localized within the ventromedial medulla suggests the crucial role of GABA/glycine inhibitory neurons in the development of RBD. It also highlights that specific brain regions can be vulnerable to the α-syn-dependent degeneration observed in RBD patients [113].

## Clinical implications

### Biomarkers

In recent years, relevant efforts have been made to develop disease-modifying therapies for PD targeting α-syn. These efforts have been limited by the difficulty in diagnosing PD accurately, especially in the early phases of the disease. Moreover, the problems in the identification of the different clinical phenotypes, as well as in the distinction of PD from other parkinsonian syndromes, have often hampered accurate patient stratification for clinical trials of therapeutic approaches targeting α-syn.

**Fig. 4:**
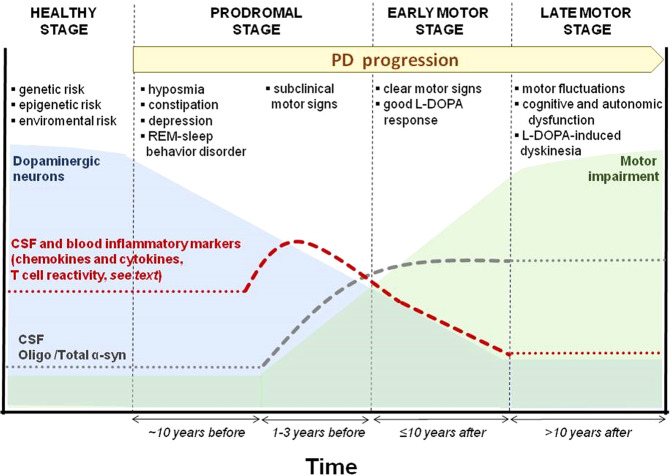
Hypothetical sequences of signs and biomarkers in PD stages. In the healthy presymptomatic stage, the number of dopaminergic cells of SNpc slowly decreases (light blue curve), and clinical symptoms are absent. In prodromal PD, neurodegeneration starts with a rapid decrease in nigral neurons survival rate (light blue), non-motor symptoms, and signs of neurodegeneration are evident; peripheral inflammatory markers begin to increase (red dashed line). Subsequently, there is an increase in inflammatory markers, which decreases gradually in the early motor stage (red dashed line). The oligo α-syn/total α-syn ratio (gray dotted line) is elevated in both prodromal and early motor stages. In addition, the evolution of the motor impairment is represented by a green curve, especially during the late motor stage, when long-term complications of dopaminergic therapy emerge (motor fluctuations and dyskinesia). The dotted lines represent a hypothetical estimation. The duration of the healthy stage is unknown, while the prodromal stage can range between 10 and 15 years.

Recently, significant progress has been made in biomarker discovery with a focus on α-syn. New assays have been employed to investigate the increasing complexity of α-syn, considering the distinct forms of this molecule existing in the biofluids and peripheral tissues during the natural history of the disease. Moreover, since growing clinical and experimental findings support the notion that PD pathogenesis begins several years before motor symptoms manifest and clinical diagnosis is made, a-syn-related biomarkers might provide a unique chance for treatment before overt massive dopaminergic denervation [114](Fig. 4). However, presymptomatic identification requires a novel methodology for objective and accurate early diagnosis. In addition, at present, no single biomarker has shown the adequate accuracy, sensitivity, and specificity required for routine clinical use. Conversely, a combination of multiple biomarkers seems to increase diagnostic accuracy [115]. Measurements of early aggregates of α-syn, such as oligomers (o-α-syn), may favor the early detection of PD since oligomerization of α-syn precedes neuronal death in the disease. For example, although several studies have reported a significantly reduced level of total α-syn (t-α-syn) in CSF of PD patients, the o-α-syn/t-α-syn ratio has been revealed to be the most sensitive and specific parameter to differentiate PD from controls [115].

Techniques such as enzyme-linked immunosorbent assay (ELISA), western blot, and mass spectrometry are used to detect t-α-syn and its oligomeric and phosphorylated isoforms in bodily fluids such as CSF, plasma, and saliva (see Table 2).

**Table 2:** Summary of techniques of synuclein species quantification in different specimens and findings in PD.

Specimen	Methods	Analyte	Diagnostic meaning
**CSF**	• ELISA, Electrochemiluminescence, xMAP technology, Single-molecule array technology	t-α-syn p-α-syn o-α-syn	↓ in synucleinopathies, ↑ as aspecific marker of synaptic damage ↑ in PD than controls, better diagnostic yield if combined with other α-syn species ↑ in PD than controls, better diagnostic yield if combined with other α -syn species
	• Real-Time Quaking-Induced Conversion (RT-QuIC), Protein-Misfolding Cyclic Amplification (PMCA)	misfolded α-syn	Can accurately detect α-syn seeding activity across the spectrum of LB-related disorders Higher seeding activity of pathologically aggregated α -syn in DLB than PD
	• Nanoscale flow cytometry	α-syn–containing extracellular vesicles (EVs)	Total α-syn–positive and aggregated α-syn–positive EV are significantly lower in patients with PD than controls
**Blood**	• ELISA	t-α-syn plasma o-α-syn plasma p-α-syn	Not conclusive results for differentiating controls from diseased groups, possible utility as a marker of disease progression ↑ in PD ↑ in PD
	• Nanoscale flow cytometry	α-syn–containing extracellular vesicles (EVs)	↑ in PD
**Saliva, tears**	• ELISA	t-α-syn o-α-syn o-α-syn/t-α-syn	Not statistically significant in distinguishing PD from controls ↑ in PD than controls, correlates with disease duration ↑ in PD than controls, increased in advanced stages
**Skin**	• Immunohistochemical analysis	t-α-syn p-α-syn	↑ in PD and synucleinopathies than tauopathies and controls Skin α-syn deposits differ in clinical variants of synucleinopathy
**Retina**	• Fluorescence immunohistochemistry	p-α-syn	in the retina of PD patients in parallel with that in the brain, including before clinical onset of the disease. It correlates with clinical disease severity
**Gut mucosa**	• Immunohistochemical analysis	t-α-syn p-α-syn	Conflicting results. More recent studies do not find significant diagnostic value in discriminating PD patients from controls
**Gonadal tissue**	• Immunohistochemical analysis	p-α-syn	Abundance of p-α-syn in the testicular and ovarian tissue of two PD patients

Novel methodological approaches to measuring protein aggregates in biological fluids have been reported, such as real-time quaking-induced conversion (RT-QuIC) and protein-misfolding cyclic amplification (PMCA). This approach detects different protein aggregates and relies on the amyloid-seeding capacity of misfolded α-syn seeds to induce recombinant native α-syn to aggregate into a measurable signal. An interesting study has cross-validated the two seeding assays, RT-QuIC and PMCA, to measure o-α-syn in the CSF of PD patients and healthy controls [116], describing reliable and reproducible results for the diagnosis of PD. A recent study using PMCA showed that the features of the α-syn aggregates in the CSF enable the differentiation of PD from MSA [117]. The authors also observed that the properties of CSF aggregates were similar to the aggregates amplified from the brain.

A growing body of evidence also highlights the importance of measuring α-syn in plasma-derived extracellular vesicles, with the possibility of isolating those of neuronal derivation [118, 119].

Non-motor symptoms such as hyposmia, urinary and gastrointestinal dysfunctions in PD patients have stimulated the search for α-syn-related biomarkers in peripheral sites and tissues implicated in these dysfunctions [120]. Materials from the skin, olfactory and gut mucosa, and salivary glands have been investigated with some important observations. However, these findings should be replicated and validated across several laboratories and large patient cohorts before reaching clinical use [121].

### Therapeutic approaches

A-syn deposition and associated immune responses may occur in different phases of the natural history of PD, the latter being an indirect effect of the former, in a vicious circle. For instance, in the prodromal stages, disturbances such as hyposmia, constipation, and depression may not be directly linked to the effect of α-syn on the nigrostriatal dopaminergic system but indirectly to other molecular processes, such as the derangement of dopaminergic circuitry other than the nigro-striatal pathway or other neurotransmitters and distinct inflammatory processes [122].

Because the corruption of α-syn contributes to defects in neurotransmission and neurodegeneration, several studies in animal models and patients have tested drugs to target α-syn (Table 3). The two main approaches are immunization and inhibition of α-syn aggregation. Immunotherapies act in two ways: active immunization, in which the immune system is stimulated to produce antibodies against α-syn, and passive immunization, in which direct administration of antibodies is provided.

**Table 3:** Clinical trials in PD patients on drugs specifically targeting α-syn via active or passive immunization or inhibiting aggregation.

Drug	NCT ID	RCT phase	Title	Start/completion date	Status	Mechanism of action	Promoter	Outcome
*Active immunization*								
AFFITOPE® PD01A	NCT01568099	Phase I	Tolerability and Safety of Subcutaneous Administration of Two Doses of AFFITOPE® PD01A in Early Parkinson’s Disease	2 April 2012–17 August 2015	Completed	Peptide-based vaccine	Affiris AG	Good tolerability and safety of subcutaneous administration (Volc et al. [123])
AFFITOPE® PD03A	NCT02267434	Phase I	Study Assessing Tolerability and Safety of AFFITOPE® PD03A in Patients With Early Parkinson’s Disease (AFF011)	17 October 2014–31 October 2016	Completed	Peptide-based vaccine	Affiris AG	Good tolerability and safety of subcutaneous administration (Poewe et al. [124])
AFFITOPE® PD01A and PD03A	NCT02270489	Phase I	Study Assessing Safety and Therapeutic Activity of AFFITOPE® PD01A and PD03A in Patients With Early MSA (AFF009)	21 October 2014–5 June 2017	Completed	Peptide-based vaccine	Affiris AG	Good tolerability and safety of subcutaneous administration (Meissner et al. [125])
UB-312	NCT04075318	Phase I A-B	Study of UB-312 in Healthy Participants and Parkinson’s Disease Patients	29 August 2019–22 February 2023	Active, not recruiting	Peptide-based vaccine	United Neuroscience Ltd.	–
*Passive immunization*								
Prasinezumab (PRX002)	NCT03100149	Phase II	Study to Evaluate the Efficacy of Prasinezumab (RO7046015/PRX002) in Participants With Early Parkinson’s Disease (PASADENA)	27June 2017–27 November 2019 (extended to 14 September 2026)	Active- not recruiting	Monoclonal antibody directed against aggregated α-syn –(C-terminus)	Hoffmann-La Roche & Prothena Biosciences Limited	Failed to meet primary endpoint, however, evidence of slower motor deterioration and consequent trial extension (Jankovic et al. [128]; Pagano et al. [129])
Prasinezumab (PRX002)	NCT04777331	Phase IIB	A Study to Evaluate the Efficacy and Safety of Intravenous Prasinezumab in Participants With Early Parkinson’s Disease (PADOVA)	5 May 2021–22 January 2024	Recruiting			–
Cinpanemab (BIIB054)	NCT03318523	Phase II	Evaluating the Efficacy, Safety, Pharmacokinetics, and Pharmacodynamics of BIIB054 in Participants With Parkinson’s Disease (SPARK)	10 January 2018–29 April 2021	Terminated	Monoclonal antibody directed against aggregated α-syn –(N-terminus)	Biogen	Failed to meet primary and secondary endpoints (Lang et al. [132])
MEDI1341	NCT04449484	Phase I	Multiple Ascending Dose Study of MEDI1341 in Patients With Parkinson’s Disease	4 August 2020–5 January 2022	Completed	Monoclonal antibody directed against all forms of α-syn –(C-terminus)	AstraZeneca	–
ABBV-0805	NCT04127695	Phase I	A Study to Evaluate the Safety and Tolerability of ABBV-0805 in Patients With Parkinson’s Disease	3 March 2020–16 June 2020	Withdrawn (strategic consideration)	Monoclonal antibody directed against aggregated α-syn	Abbvie	Good safety and tolerability in healthy subjects (Kalluri et al. 2021)
Lu AF82422	NCT03611569	Phase I	Lu AF82422 in Healthy Non-Japanese and Japanese Subjects and in Patients With Parkinson’s Disease	25 July 2018–26 July 2021	Completed	Monoclonal antibody directed against all forms of α-syn –(C- and N- terminus)	H. Lundbeck A/S	–
Lu AF82422	NCT05104476	Phase I	A Study of Lu AF82422 in Participants With Multiple System Atrophy (AMULET)	10 November 2021–30 August 2023	Recruiting			–
*Aggregation inhinibtion*								
Anle138b	NCT04685265	Phase I	A Study to Assess the Safety, Tolerability, Pharmacokinetics and Pharmacodynamics of anle138b in Parkinson’s Disease	22 December 2020–December 2022	Recruiting	General inhibitor of protein aggregation	MODAG GmbH	–
UCB0599 (NPT200-11)	NCT04875962	Phase Ib	Phase 1 Study to Evaluate the Safety and Tolerability UCB0599 in PD patients	6 May 2019–19 February 2020	Completed	Inhibitor of α-syn misfolding and aggregation	Neuropore Therapies Inc.; Novartis Pharmaceuticals Corporation, UCB S.A	Good safety and tolerability in both HC and PD patients (extension study ongoing) (Smit et al. [136])
UCB0599 (NPT200-11)	NCT04658186	Phase II	Study to Evaluate the Efficacy, Safety, Tolerability and Pharmacokinetics of Oral UCB0599 in Participants With Early stage PD	30 December 2020–18 July 2023	Recruiting			–

Concerning active immunization against α-syn, AFFiRiS (AC Immune recently acquired the α-syn antibody program), developed 2 peptide vaccines, AFFITOPE® PD01A and PD03A. The first human phase 1 randomized study recruited subjects with early PD and MSA for both drugs. Patients randomly received vaccinations of low- or high-dose PD01A or PD030A, respectively. Both doses of the drugs were well tolerated. Both vaccines showed a clear immune response against α-syn targeted epitopes [123–125]. Another synthetic peptide-based vaccine, UB-312, is currently being tested in healthy controls and early PD patients.

Regarding passive immunization against α-syn, encouraging preclinical findings were published showing a reduction in α-syn aggregates in the brain, associated with decreased motor and cognitive alterations [126, 127]. Roche (Basel, Switzerland) and Prothena (Dublin, Ireland) conducted a multicenter, randomized, double-blind phase 2 trial in patients with early PD to evaluate the efficacy of Prasinezumab (PRX002) vs. placebo, which failed to meet the primary endpoint [128, 129]. However, the study was extended until September 2026 since a post-hoc analysis revealed a slower disease progression in the treated arm. Moreover, another multicenter, randomized, double-blind phase 2B trial is ongoing to evaluate the efficacy of Prasinezumab in slowing disease progression. Currently, the safety and pharmacokinetic profiles of two other drugs, MEDI1341 and lu-AF82422 are being studied in phase I trials. Abbvie, instead, withdrew ABBV-0805 phase I trial for strategic considerations, although it was shown to have good tolerability in healthy subjects [130]. Cinpanemab (BIIB054) from Biogen (Cambridge, MA), is another monoclonal antibody that targets the N-terminal of α-syn. A phase I study in healthy controls and PD patients showed Cinpanemab favorable safety, tolerability, and pharmacokinetic profiles [131]. However, the subsequent phase II trial was terminated because Cinpanemab did not improve scores on the motor rating scale [132]. It is unknown if Cinpanemab reduced the abundance of α-syn aggregates. Indeed, it is possible that N- and C-terminal epitopes are differentially exposed in aggregated α-syn, as demonstrated in vitro [133]. This study highlights the need for biomarkers of aggregated α-syn to determine if putative therapies engage α-syn.

A promising line of research is the development of oligomer modulators. Among these drugs, an aggregation inhibitor, anle138b, has been first investigated. In in vitro and in vivo experiments, anle138b blocked the formation of pathological aggregates of prion protein and of α-syn and inhibited oligomer accumulation, neuronal degeneration, and disease progression [134]. Evotec (Nottingham, UK) is currently conducting a double-blind, placebo-controlled, multiple ascending dose phase I study in patients with mild to moderate PD. Another drug, UCB0599 (aka NPT200-11), inhibits misfolding and aggregation of normal α-syn [135], showed a good safety and tolerability profile, and is currently being tested by Neuropore Therapies Inc (San Diego, CA), in phase 2 clinical trial in early PD patients [136].

As shown in Table 3, none of the molecules tested in a phase II trial have yet met their primary endpoint. This could have several possible explanations. First of all, the timing: the degenerative processes start years before the onset of symptoms. Therefore, patients included in trials could have initiated the treatment too late, once most of the damage had already occurred. Secondly, α-syn aggregation and spreading are one but not the only pathogenic mechanisms underlying PD initiation and progression. As a consequence, it could not be enough to target only one of these factors, but a combination of therapeutic approaches with different targets could be more powerful. Finally, the trial design could not have been appropriate to highlight, for example, a slowing of symptoms progression in early PD patients for too short follow-up or a wrong choice of primary endpoint measures.

Furthering our understanding of how abnormal α-syn impacts neurotransmission could also help develop new therapeutics. For example, targeting metabotropic receptors or transporters that control the release of neurotransmitters that are perturbed early in PD could prevent neurodegeneration. Targeting genes that impact α-syn transport and turnover, such as *LRRK2* and *GBA* could also show promise in preventing the progression of PD.

## Conclusions

More than twenty years of investigation on the role of α-syn in PD and the development of potential strategies to target this molecule to cure the disease represent a long story. It might somehow recall to our mind the long journey that has represented the story of the discovery of the central role of DA in PD and the subsequent development of successful symptomatic DA-based therapies. It is also possible that targeting only α-syn might not be sufficient to modify the natural history of the disease, and other molecular culprits must be identified and targeted. Similarly, we now know that efforts should be made to identify α-syn-related precocious mechanisms contributing to the initiation and propagation of the disease, such as synaptopathy and inflammation.

## Search strategy and selection criteria

We searched PubMed between November 2020 and August 2022 for articles published in English from 11 Nov 1983, to 31 August 2022. We further examined the reference lists from relevant articles. A combination of keywords related to alpha-synuclein, synaptic plasticity and Parkinson’s disease was used: “alpha-synuclein”, “neuroinflammation”, “neurodegeneration”, “Lewy bodies”, “Parkinson’s disease”, “striatal dopaminergic transmission”, “synucleinopathies”, “synaptopathy”, “synaptic plasticity”, “presynaptic”, “postsynaptic, “toxicity”, “misfolded”, “aggregation”, “oligomers”, “fibrils”, “animal models”, “mechanisms”, “Lewy body dementia”, “multiple system atrophy”, “pure autonomic failure”, “REM behavior disorder”. We then selected the most relevant papers with particular attention to studies dealing with alpha-synuclein earliest pathological effects published within the past 5 years, although seminal older studies were included for their importance. The final reference list was generated on the basis of relevance to the topics covered in this review. Clinical trials in the Table 3 were identified through searches in ClinicalTrials.gov, a resource provided by the U.S. National Library of Medicine, using the term “alpha-synuclein”. We applied the following filters to search results: “not yet recruiting”, “recruiting”, “active, not recruiting”.

## Data Availability

Data sharing not applicable to this article as no datasets were generated or analyzed during the current study.
